# An Insertable Passive LC Pressure Sensor Based on an Alumina Ceramic for *In Situ* Pressure Sensing in High-Temperature Environments

**DOI:** 10.3390/s150921844

**Published:** 2015-08-31

**Authors:** Jijun Xiong, Chen Li, Pinggang Jia, Xiaoyong Chen, Wendong Zhang, Jun Liu, Chenyang Xue, Qiulin Tan

**Affiliations:** 1 Key Laboratory of Instrumentation Science & Dynamic Measurement, Ministry of Education, North University of China, Tai Yuan 030051, China; E-Mails: xiongjijun@nuc.edu.cn (J.X.); wdzhang@nuc.edu.cn (W.Z.); liuj@nuc.edu.cn (J.L.); 2 Science and Technology on Electronic Test & Measurement Laboratory, North University of China, Tai Yuan 030051; E-Mails: pgjia@nuc.edu.cn (P.J); chenxiaoyong@nuc.edu.cn (X.C.); xuechenyang@nuc.edu.cn (C.X.)

**Keywords:** passive LC pressure sensor, Alumina ceramic, Pressure measurement, High-temperature application

## Abstract

Pressure measurements in high-temperature applications, including compressors, turbines, and others, have become increasingly critical. This paper proposes an implantable passive LC pressure sensor based on an alumina ceramic material for *in situ* pressure sensing in high-temperature environments. The inductance and capacitance elements of the sensor were designed independently and separated by a thermally insulating material, which is conducive to reducing the influence of the temperature on the inductance element and improving the quality factor of the sensor. In addition, the sensor was fabricated using thick film integrated technology from high-temperature materials that ensure stable operation of the sensor in high-temperature environments. Experimental results showed that the sensor accurately monitored pressures from 0 bar to 2 bar at temperatures up to 800 °C. The sensitivity, linearity, repeatability error, and hysteretic error of the sensor were 0.225 MHz/bar, 95.3%, 5.5%, and 6.2%, respectively.

## 1. Introduction

There is a growing demand for measurement of pressure in harsh environments, especially in high-temperature environments, such as are found in automotive, turbine, aerospace, and industrial applications [1,2,3,4]. For example, the air-pressure monitoring and control for the combustor of the turbine engine can help to reduce the risk of stall, and this requires a pressure sensor that can operate above 600 °C for *in situ* pressure measurement. Therefore, research on pressure sensors that can perform reliably in high-temperature environments has become increasingly important. Traditional pressure sensors are based on silicon or silicon-on-insulator materials, and they do not function in high-temperature environments because the electric wire and pressure sensitive structure impair functionality at temperatures above 300 °C [5,6,7,8]. Although silicon carbide and gallium nitride materials permit pressure measurements in moderately high-temperature environments, pressure sensors capable of operation at temperatures above 600 °C have yet to be reported [9,10]. Furthermore, most high-temperature pressure sensors require a battery power supply, which increased the complexity of the sensor. Passive wireless sensors have characteristics of having no batteries and contactless signal readout, which have extensive application prospects and can conduct *in situ* measurement in many fields, such as hermetic space, humidity monitoring, and high-temperature measurement. 

Some wireless passive LC sensors using low-temperature co-fired ceramic (LTCC) or high-temperature co-fired ceramic (HTCC) technology have been developed to conduct *in situ* pressure, temperature, and other parameter measurements in high-temperature environments. The properties of these ceramic materials are able to ensure stable operation of the sensor in high-temperature environments, and their resonant signals can be read wirelessly through the magnetic coupling between the passive sensors and reader antennae. However, higher temperatures greatly influence these ceramic pressure sensors, especially their inductance coils, producing greater resistance in the sensors and a lower quality factor. Since the quality factor of a passive LC sensor describes the relationship between energy storage and energy consumption, a higher quality factor is beneficial for the acquisition of power via inductive coupling, as well as for transmitting data to an external receiver. Because of the lower quality factor of the LTCC and HTCC sensors in high-temperature environments, the coupling strength is very weak between the sensor and antenna, impeding wireless signal transmission and precluding precise detection of pressure signals. Moreover, the LTCC material cannot be utilized at temperatures over 600 °C, and the LTCC sensor cannot operate at temperatures above 600 °C. For example, in 2002, the Georgia Institute of Technology first proposed a wireless passive LTCC high-temperature pressure sensor that demonstrated a sensitivity of up to 150 kHz/bar; however, the sensor could only operate at temperatures up to 450 °C [11,12]. Subsequently, Radosavljevic and Xiong *et al.* proposed improved LTCC high-temperature pressure sensors that showed significant improvement in sensitivity and pressure testing range, respectively; however, the operating temperatures could not exceed 600 °C [13,14,15,16]. In 2013, some HTCC high-temperature pressure sensor were proposed, in which the material withstood temperatures exceeding 800 °C, with a coupling distance of up to 2.8 cm; however, the sensors can only be tested below 600 °C [17,18,19]. In 2014, Li and Zhang proposed an integrated LC ceramic pressure sensor respectively, which can be tested up to 800 °C, but higher temperature greatly influence the inductance of the LC sensor, producing a lower quality factor and imprecise measurements results [20,21]. Recently, Boccard *et al.* proposed a new approach to measure temperature in the far-field region using a dielectric resonator, which the operated temperature can be up to 700 °C, and the working distance can be up to 1.06 m. However, the pressure sensors based on this concept for high temperature application have not been reported yet [22].

This paper presents a wireless passive LC pressure sensor based on an alumina ceramic. The inductance and capacitance elements of the sensor were designed separately, and then independently integrated on two ceramic substrates using thick film integrated technology, which can produce a sensor operational in high-temperature environments with a high quality factor. The sensor was then tested in a high-temperature pressure system, and the accuracy of the pressure characterization of the sensor in high-temperature environments was investigated.

## 2. Measurement Principle and Design of the Sensor

**Figure 1:**
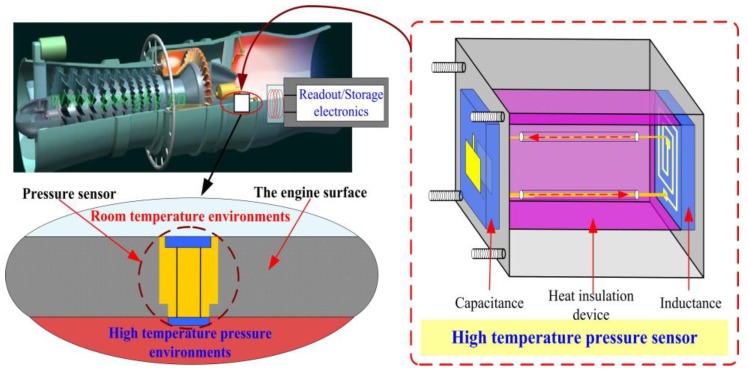
Proposed high-temperature pressure monitoring method in a practical application.

Figure 1 presents the proposed high-temperature pressure monitoring method in a practical application. In a working engine, the sensor would be embedded in the engine surface, with the capacitance element of the sensor internal to the engine, conducting *in situ* real-time pressure monitoring, and the inductance element of the sensor is external to the engine, magnetically coupled with the readout/storage electronics to facilitate pressure signal data collection and storage. The sensor consists of an inductance coil and a capacitance plate, which form a series LC resonant circuit. The resonant frequency *f_0_* and quality factor *Q* of the sensor are expressed as follows:

(1) $$f_{0}=1/(2\pi \sqrt{L_{S}C_{S}})$$

(2) $$Q=(1/R_{s})(\sqrt{L_{s}/C_{s}})$$

where *L*_s_, *C*_s_, and *R*_s_ are the inductance, capacitance, and resistance of the sensor, respectively. 

**Figure 2:**
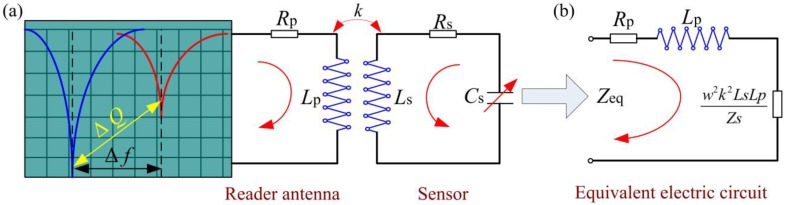
(**a**) Wireless measurement principle; (**b**) Equivalent electric circuit.

The pressure can be wirelessly detected by measuring the change in the sensor’s resonant frequency, according to the measurement principle shown in Figure 2a, in which *L*_p_ and *R*_p_ are the inductance and resistance of the reader antenna, respectively.

Figure 2 shows the equivalent circuit of the input impedance as viewed from the reader antenna, the parameters *R*_p_ and *L*_p_ represent inductance and resistance of the reader antenna, *k* is the coupling coefficient between the sensor and the reader antenna, and the parameters *R*_s_, *L*_s_ and *C*_s_ represent inductance, resistance and capacitance of the sensor. In addition, *w* and *Z*_s_ are the angular frequency and impedance of the sensor. According to Kirchhoff's law and the theory underlying transformer operation, the equivalent impedance *Z_eq_* of the sensor is defined as [23,24,25]:

(3) $$Z_{eq}=R_{p}+j2\pi f_{0}L_{p}[1+\frac{k^{2}(f/f_{0})}{1-{\left(f/f_{0}\right)}^{2}+jf/(f_{0}Q)}]=F(f_{0})$$

where *f* is the excitation frequency of the reader antenna. From Equation (3), the input impedance phase *I_m_(Z)* can be expressed as follows:

(4) $$I_{m}(Z)=2\pi fL_{p}[1-{\left(f_{0}/f\right)}^{2}+k^{2}Q^{2}\frac{1-{\left(f/f_{0}\right)}^{2}}{1+Q^{2}{\left(f/f_{0}-f_{0}/f\right)}^{2}}]$$

When a bandwidth frequency sweep signal is loaded on the reader antenna, and a certain frequency of the sweep is approximately equal to the resonant frequency of the sensor *f*_0_, the passive sensor is excited at resonance, and the impedance phase will produce a significant change in the input impedance phase. From Equation (4), we can conclude that the input impedance phase *I*_m_(Z) is a function of the resonant frequency of the sensor, and thus the resonant frequency of the sensor can be detected accurately by monitoring the change of the impedance phase *I*_m_(Z). Furthermore, the pressure characterization of the sensor can then be obtained indirectly by analyzing the variation in the sensor’s resonant frequency *f*_0_ in high-temperature environments. 

**Figure 3:**
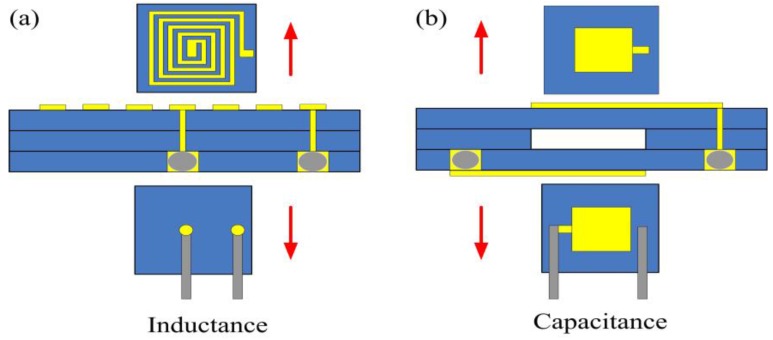
Design schematic of the (**a**) inductance and (**b**) capacitance elements of the sensor.

From Equation (2), we can conclude that at increasing temperatures, increasing resistance in the inductance coil will induce a significant decrease in the quality factor, which is not conducive to signal transmission in high-temperature environments. However, if the inductance and capacitance elements of the sensor are separated by a thermal insulation material, the inductance and capacitance elements operate in room-temperature environments and high-temperature environments, respectively. In addition, when the sensor insert in high temperature environments for pressure measurement, the inductance coil of the sensor will have little effect on the quality factor of the sensor. Figure 3a,b show designs of the inductance and capacitance elements, respectively, each containing of three layers of alumina ceramic tapes; these inductance and capacitance elements can be connected with silver (Ag) wire to form a passive series LC sensor. 

The inductance design includes a square spiral inductor, the dimensions of which can be calculated as follows [26]:

(5) $$Ls=0.00000296\frac{n^{2}(\frac{d_{out}+d_{in}}{2})}{1+2.75(\frac{d_{out}-d_{in}}{d_{out}+d_{in}})}$$

where *n* is the number of inductor coils, *d*_in_ is the inner diameter of the inductor coil, and *d*_out_ is the outer diameter of the inductor coil. The square plate of the capacitance element can be calculated as follows:

(6) $$Cs=\frac{\varepsilon_{0}a^{2}}{t_{g}+\left(2t_{m}/\varepsilon_{r}\right)}\cdot \frac{{\text{tanh}}^{-1}(\sqrt{d_{0}/\left(t_{g}+(2t_{m}/\varepsilon_{r})\right)})}{\sqrt{d_{0}/\left(t_{g}+(2t_{m}/\varepsilon_{r})\right)}}$$

(7) $$d_{0}=\frac{3Pa^{4}(1-v^{2})}{16E{\left(t_{m}\right)}^{3}}$$

where *ε*_0_ is the vacuum dielectric constant, *ε*_r_ is the relative permittivity of the alumina ceramic material, *t*_g_ is the height of the embedded cavity, *t_m_* is the thickness of the sensitive membrane, *E* is Young’s modulus, *v* is Poisson’s ratio, *P* is the atmospheric pressure outside the sensor, and *a* is the side length of the capacitance plate. The specific LC structure design parameters are shown in Table 1.

**Table 1:** Geometric structural parameters of the sensor.

Symbol	Parameters	Value
*d*_in_	Inner diameter of the spiral inductor	~8 mm
*d*_out_	Outer diameter of the spiral inductor	~50 mm
*n*	Number of turns of the inductor coil	10
*a*	Side length of the capacitance plate	~12 mm
*t*_g_	Height of the sealed cavity	200 µm

## 3. Fabrication Procedure

**Figure 4:**
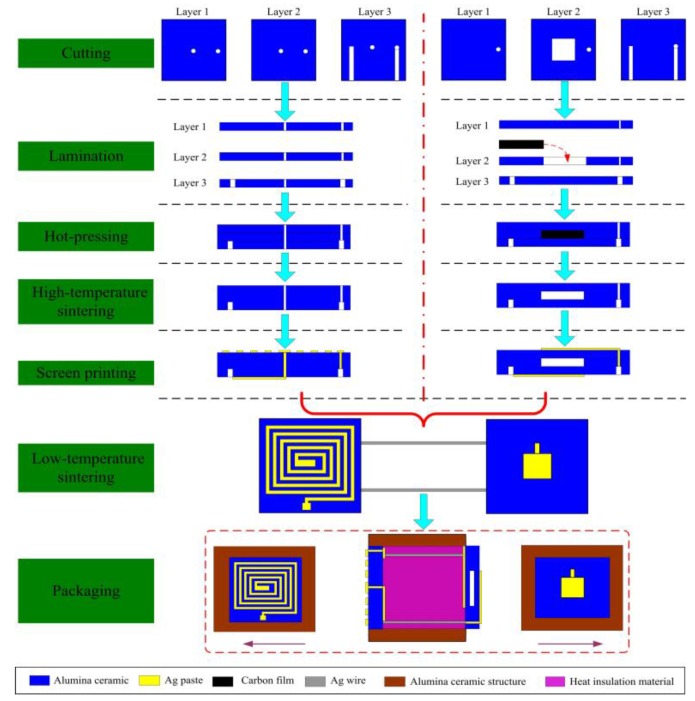
Fabrication process.

**Figure 5:**
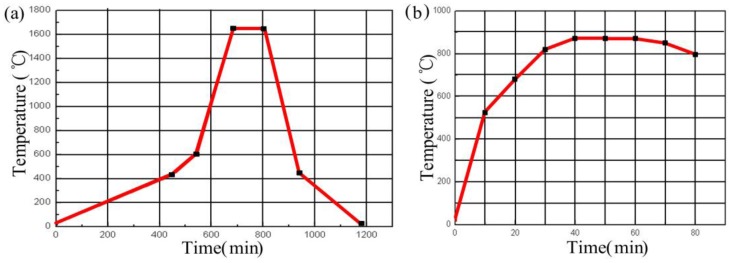
Sintering curve for the curing process of (**a**) the alumina ceramic substrates and (**b**) the electrical elements.

**Figure 6:**
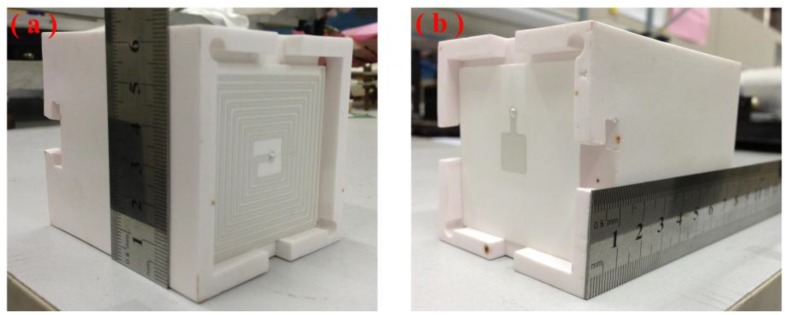
Image of the (**a**) inductance and (**b**) capacitance of the sensor sample.

Alumina green tapes, Ag wire, and conductive Ag ink were used to fabricate the sensor. In addition, the melting temperature of Ag is above 960 °C, which ensure the feasibility of the sensor in high temperature environments. The sensor was monolithically fabricated, as depicted in Figure 4, which included cutting, drilling, filling, laminating, hot-pressing, high-temperature sintering, screen-printing, low-temperature sintering, and packaging, and associated processing steps. The inductance and capacitance structures each consist of three layers of alumina green tapes, which were placed in a drying oven at 80 °C for 30 min. In the inductance element, Layer 2 was the same as Layer 1. In the capacitance element, Layer 2 was cut using the NDYAG micromachining laser system to produce with accuracy a cavity matching the designed punch file. A carbon film was then manufactured with the same punch file used to produce the diaphragm of the chamber, and the chamber was lined with the carbon film, which would volatilize to form a sealed cavity during co-firing. In the conductance and inductance elements, Layer 3 consisted of one sheet of ceramic green tape, in which two channels were cut using a different punch file. In both elements, all three layers were stacked together under specific temperature and pressure conditions, bonding them tightly to form the two ceramic substrates. These stacks were sintered in a box furnace to cure the ceramic substrates; the specific sintering curve is shown in Figure 5a. After firing, the respective plate and coil of the capacitance and inductance elements were fabricated using screen-printing technology, and then the Ag wire was placed in the channels in the ceramic substrates to establish connections between the two elements. The connected ceramic substrates were then sintered in a furnace at a peak temperature of 875 °C and a total firing time of approximately 80 min to allow the Ag ink to cure, forming an LC sensor. The sintering curve for this step is shown in Figure 5b. After the low-temperature sintering, the sensor was integrated on a thermally insulating structure that separated the two elements by a certain distance, to allow the capacitance and inductance elements to operate in high-temperature environments and room-temperature environments, respectively. A digital image of the final fabricated ceramic sensor sample is shown in Figure 6.

## 4. Experimental Procedure and Results

**Figure 7:**
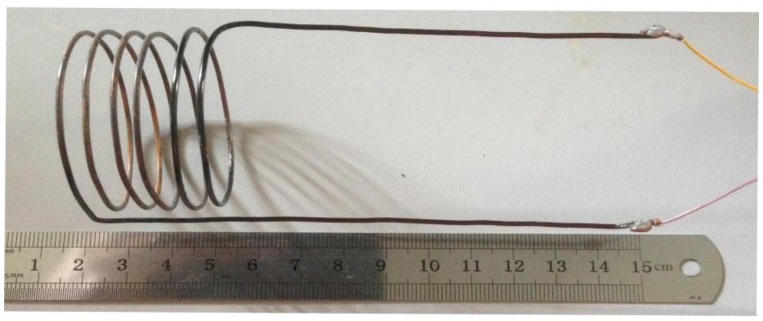
Reader antenna.

The testing antenna was fabricated of copper, which has excellent mechanical properties and electrical performance. As shown in Figure 7, the antenna’s outer diameter was 31.6 mm, and it contained 5.5 turns at an average distance from one another of 5.45 mm. The self-resonant frequency of the antenna was approximately 61.3 MHz, and the input impedance phase can be read in an effective bandwidth range between 1 MHz and 61.3 MHz. 

**Figure 8:**
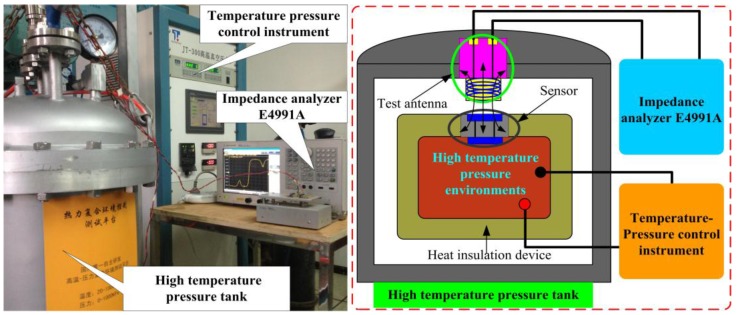
High-temperature pressure measurement system.

**Figure 9:**
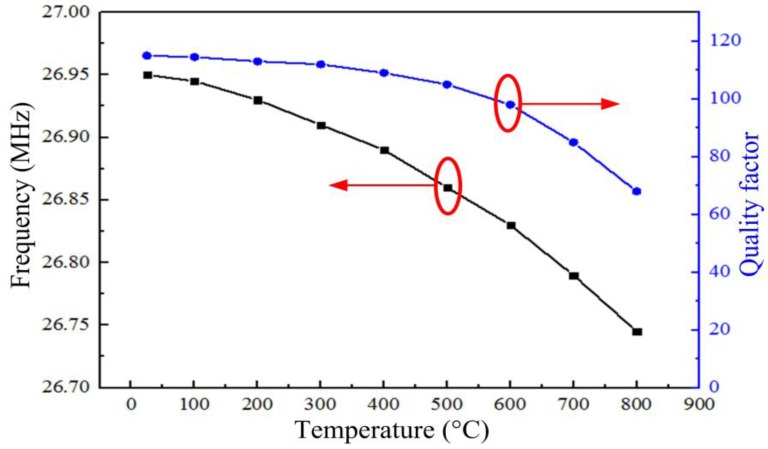
Resonant frequency *f*_0_ and quality factor *Q* of the sensor as a function of temperature.

To investigate the pressure characterization of the fabricated sensor in high-temperature environments, the sensor’s performance was measured as a function of temperature and pressure using a high-temperature pressure measurement system, consisting of an E4991A impedance analyzer, a high-temperature pressure tank, and a temperature pressure control instrument, as shown in Figure 8. The temperature pressure control instrument allows accurate control of temperatures from room-temperature to 800 °C and pressures from 0 bar to 2 bar. When the sensor is placed in the system’s high-temperature pressure tank, the capacitance element of the sensor can operate up to 800 °C for *in situ* pressure monitoring, and the inductance element of the sensor operates below 200 °C for pressure signal output. Ultimately, the *in situ* pressure signal in the high-temperature pressure tank was captured wirelessly by the E4991A impedance analyzer through the reader antenna coupled with the sensor. A set of high-temperature pressure experiments on the sensor in the high-temperature pressure testing tank were carried out by varying the temperature and pressure to investigate the sensor’s high-temperature response. The resonant frequency and quality factor of the sensor as a function of temperature are shown in Figure 9.

Figure 9 shows that the resonant frequency of the sensor at room-temperature was approximately 26.95 MHz, its resonant frequency at 800 °C was 26.75 MHz, and the average slope of the resonant frequency from room-temperature to 800 °C was approximately −0.25 kHz·°C^−1^. The quality factor of the sensor reduced from 110 at room-temperature to 68 at 800 °C, the change is smaller than the sensor proposed by Zhang in 2014 [21].

**Figure 10:**
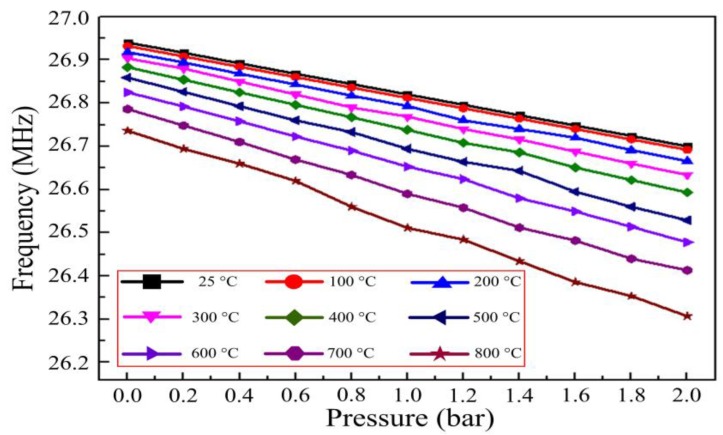
Resonant frequencies of the sensor as a function of pressure at varying temperatures.

Figure 10 shows the resonant frequencies of the sensor when different pressures were applied to it at elevated temperatures, indicating that as pressure increased the resonant frequency decreased linearly in elevated temperature environments. Furthermore, the sensitivity of the sensor increased as the temperature increased, from 0.11 MHz/bar at room-temperature to 0.225 MHz/bar at 800 °C.

**Figure 11:**
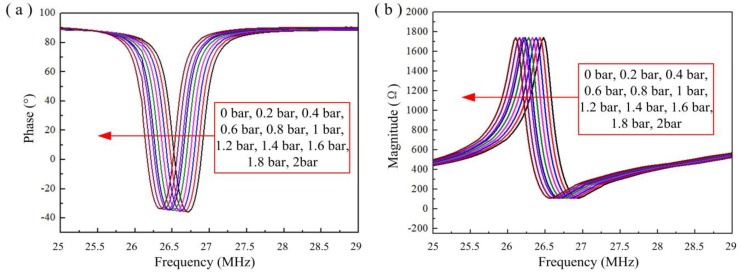
Impedance (**a**) phase and (**b**) magnitude, in response to different pressures at 800 °C.

**Figure 12:**
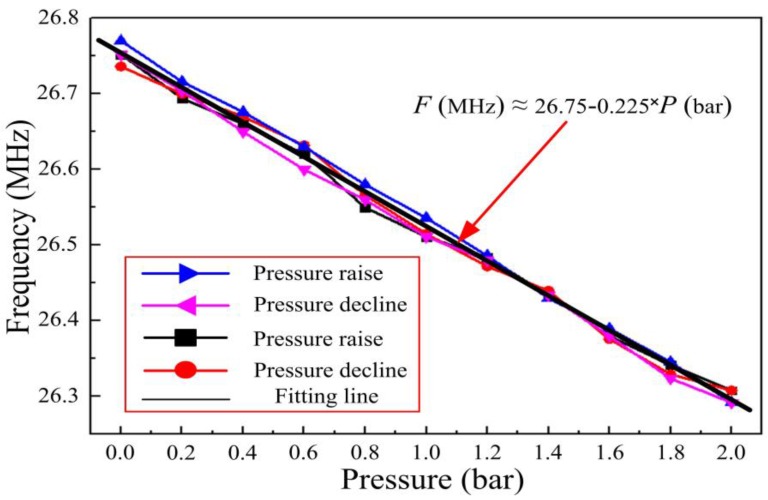
The resonant frequency of the sensor as a function of pressure at 800 °C.

Figure 11 shows the sensor’s impedance phase and magnitude as a function of pressure at 800 °C. Figure 11 demonstrates effective coupling between the sensor and reader antenna at 800 °C, and the resonant frequency of the sensor can be obtained from the phase minimum of each curve. Many repetitions of pressure experiments at 800 °C were carried out to investigate the pressure characterization of the sensor at 800 °C, and the specific test results are shown in Figure 12.

From Figure 12, we can see that the linearity, repeatability error, and hysteretic error of the sensor were 95.3%, 5.5%, and 6.2%, respectively, as a function of pressure at 800 °C. Curve fitting produces a frequency-pressure relationship of the sensor at 800 °C that can be expressed as *F* (MHz) = 26.75–0.225 *P* (bar). Therefore, the pressure sensitivity of the sensor at 800 °C is approximately 0.225 MHz/bar.

## 5. Conclusions

This work successfully demonstrates a design and fabrication method for an implantable high-temperature passive LC ceramic pressure sensor operable at temperatures up to 800 °C. The inductance and capacitance elements of the sensor were designed separately and installed at a distance from one another, separated by a thermally insulating material, allowing the sensor to operate in high-temperature environments with a high quality factor. The sensor was fabricated using thick film integrated technology based on alumina ceramic material, further ensuring operational stability in high-temperature environments. The sensor was evaluated in a high-temperature pressure testing system, and the experimental results showed that the sensor is capable of conducting pressure measurements from 0 bar to 2 bar at temperatures up to 800 °C, with a sensitivity, linearity, repeatability error, and hysteretic error of 0.225 MHz/bar, 95.3%, 5.5%, and 6.2%, respectively. The pressure range for the turbine engine application is above 1 Mpa, which the sensor is far from meeting the requirements. Future work will be focused on improving the pressure range of the sensor for practical application.
